# Animal Models to Study Links between Cardiovascular Disease and Renal Failure and Their Relevance to Human Pathology

**DOI:** 10.3389/fimmu.2015.00465

**Published:** 2015-09-17

**Authors:** Tim D. Hewitson, Stephen G. Holt, Edward R. Smith

**Affiliations:** ^1^Department of Nephrology, Royal Melbourne Hospital (RMH), Melbourne, VIC, Australia; ^2^Department of Medicine – RMH, University of Melbourne, Melbourne, VIC, Australia

**Keywords:** animal models, cardiorenal syndrome, heart, kidney, review

## Abstract

The close association between cardiovascular pathology and renal dysfunction is well documented and significant. Patients with conventional risk factors for cardiovascular disease like diabetes and hypertension also suffer renal dysfunction. This is unsurprising if the kidney is simply regarded as a “modified blood vessel” and thus, traditional risk factors will affect both systems. Consistent with this, it is relatively easy to comprehend how patients with either sudden or gradual cardiac and or vascular compromise have changes in both renal hemodynamic and regulatory systems. However, patients with pure or primary renal dysfunction also have metabolic changes (e.g., oxidant stress, inflammation, nitric oxide, or endocrine changes) that affect the cardiovascular system. Thus, cardiovascular and renal systems are intimately, bidirectionally and inextricably linked. Whilst we understand several of these links, some of the mechanisms for these connections remain incompletely explained. Animal models of cardiovascular and renal disease allow us to explore such mechanisms, and more importantly, potential therapeutic strategies. In this article, we review various experimental models used, and examine critically how representative they are of the human condition.

## Introduction

Patients with conventional risk factors for cardiovascular disease (CVD), like diabetes and hypertension, also suffer renal dysfunction. This is unsurprising if the kidney is simply regarded as a “modified blood vessel” with traditional risk factors likely to affect both systems and coexist (1). However, although a problem in one organ system affects the other, the prime mover in this loop may be occult. Animal models have helped us tease apart these associations but have consistently shown that impairment of one organ has detrimental effects on the other at functional, biochemical, and molecular level [reviewed in Ref. (2–5)]. First described by El-Atat et al. (6), these clinical interactions are now collectively known as the cardiorenal syndromes (CRS), which for many years were considered a single diagnostic group, and thus, may have limited or confounded many early mechanistic studies.

**Figure 1:**
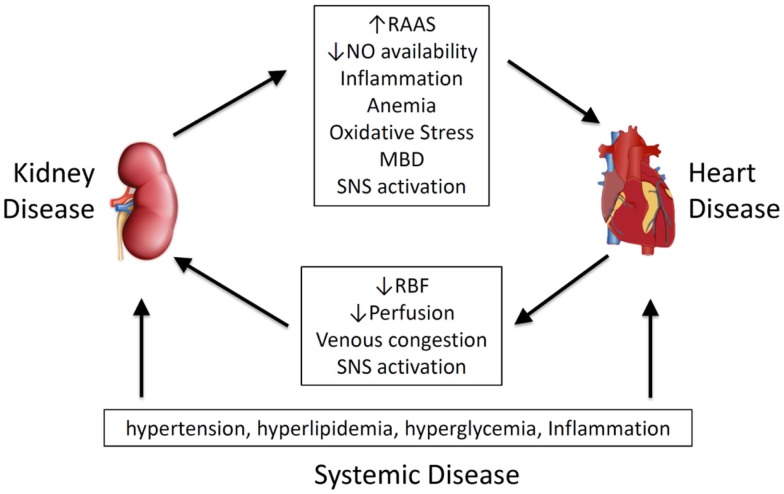
**Pathophysiological mediators of cardiorenal syndrome and renocardiac syndrome**. MBD, mineral bone disorder; NO, nitric oxide; RAAS, renal–angiotensin–aldosterone system; RBF, renal blood flow; SNS, sympathetic nervous system.

Given its importance, and the wide spectrum of primary disease and clinical presentation, a number of classification systems have been proposed. Ronco et al. have classified CRS on time frame (acute, chronic or secondary) and which organ is involved first (heart or kidney) recognizing five different types of the CRS or renocardiac syndromes (RCS) (7, 8). Other approaches are based on pathophysiological links (9–11) (Figure 1), arguing that effects are bidirectional (11), and that temporal differences in organ involvement are artificial (9).

As the natural history is sometimes slow and consequently difficult to explore in clinical trials, adequate experimental modeling of the clinical scenario is crucial to examining mechanisms and potential therapeutic strategies. In this review, we use the Ronco classification (7) to discuss animal approximations of the CRS.

## Models of Cardiac Injury Causing Renal Dysfunction (Types 1/2)

Neurohumoral mechanisms have evolved to maintain a relatively constant blood volume and organ perfusion under continuously changing conditions (11). In the context of a failing pump (heart), vasopressor systems, like the sympathetic nervous system (SNS) and renin–angiotensin–aldosterone system (RAAS), are activated to maintain the hemodynamic balance (12). Vasoconstriction of the efferent artery helps maintain glomerular filtration rate (GFR) in low-output states, but the increased vascular resistance may reduce overall renal perfusion and cause intra-renal hemodynamic changes. This can occur acutely in the setting of abrupt hypotension [e.g., due to myocardial infarction (MI)] or chronically, which over time causes tubular hypoxia and apoptosis leading to a loss of nephron mass and function.

In animal models, MI, produced by ligation of the left anterior descending coronary artery has detrimental effects on the renal function over time. GFR significantly decreases after MI within 4 weeks, and deteriorates further at 16 weeks. Histological analysis reveals greater renal interstitial fibrosis and downstream transforming growth factor-β1 (TGFβ1) signaling (smad2 phosphorylation) at all time-points (13).

In rodents, beta-adrenergic stimulation through multiple isoproterenol injections results in left ventricular (LV) fibrosis (14). It remains unclear if this interference with the SNS has renal consequences, consistent with CRS.

Hemodynamic changes are a well-recognized driving force in the pathophysiology of CRS. A reduction in cardiac output (forward failure) reduces renal blood flow leading to changes in distal tubular chloride and other solute content, resulting in increased renin release from the macula densa and activation of the RAAS (15). Surgical restriction of the carotid artery leads to aortic regurgitation (16) with both cardiac hypertrophy and albuminuria from 5 months (17).

Higher central venous pressures due to congestion may increase both interstitial pressure and efferent pressure (through a decreased afferent-efferent gradient) (5). In these circumstances, elevated venous pressures (backward failure) may reduce renal blood flow and consequently urine output more than a reduction in arterial pressure, ultimately leading to hypoxia and activation of the RAAS. Elevated renal venous pressure alone reduces renal arterial flow and GFR in the pig, with increases in plasma renin activity (18). While maintaining cardiac homeostasis initially, long-term activation of the RAAS eventually leads to progression and myocardial remodeling through fibrosis (3).

## Models of Acute/Chronic Renal Disease Causing Cardiac Dysfunction (Type 3/4)

As chronic kidney disease (CKD) progresses, the SNS is stimulated as a result of renal ischemia, activation of the RAAS and suppression of nitric oxide (NO) synthesis. This results in hypertension, left ventricular hypertrophy (LVH), and progressive LV dilatation (12). Early cardiac hypertrophy, and subsequent fibrosis due to fibroblast activation (19, 20), are final common contributors to organ dysfunction irrespective of the nature of the initial injury.

### Cardiac pathologies in renal models

Sub-total nephrectomy, often termed 5/6 nephrectomy, is probably the most established method of modeling progressive renal failure seen with loss of renal mass. Although commonly performed in the rat, similar rabbit and mouse models exist but appear less reliable. Rather than mimicking a renal disease *per se*, sub-total nephrectomy parallels the consequences of reducing functional nephron number. The predominant pathological abnormalities within the remaining kidney are glomerulosclerosis and tubulointerstitial fibrosis (21). It is important to appreciate that two quite different models are encompassed by the expression “5/6 nephrectomy”, namely an ablation model widely used for the study of CRS and a less common ligation model. In the former, one kidney is removed along with ~50% of the contralateral kidney by polar excision 1–2 weeks later, with the rate of renal decline very closely related to the amount of tissue excised (21). In contrast to human chronic kidney disease (CKD), although LVH is a consistent feature (22) and resembles that seen in early human CKD (23), severe hypertension is not usually a feature of this model. Nevertheless early LV diastolic dysfunction is seen with commensurate increases in heart weight (corrected for body weight), and myocyte cross sectional area (24, 25). Other documented pathologies include increased myocardial artery wall thickness, capillary density (26) and interstitial fibrosis (24). Similar changes are seen in mice, but tend to be more strain dependent (4).

#### Modeling CKD-Specific Risk Factors

While traditional cardiovascular risk factors are highly prevalent in patients with CKD, results of clinical trials focusing on controling such factors have been largely disappointing, and thus, non-traditional risk factors associated with CKD are being increasingly explored.

##### Anemia

Anemia is a feature of most CKD and related to erythropoietin deficiency (27), yet it is unclear whether it is a mediator of CRS or simply a marker of disease progression (11). There is some experimental evidence that correction of anemia preserves both renal and cardiovascular function. Cardiomyopathy occurs after IV injection of anthracycline antibiotics, such as adriamycin (28, 29), with renal fibrosis and pronounced loss of renal function accompanying LV enlargement (29). In the doxorubicin model administration of darbepoetin can increase Hb to control levels and significantly attenuates the renal dysfunction, renal interstitial fibrosis and LV weight (29). Likewise, erythropoietin improves cardiac function post-MI, an effect seemingly related to the promotion of neovascularization (30). Effects in a combined cardiac-renal model have not been examined.

##### Inflammation

Progressive renal impairment is characterized by a chronic inflammatory state with elevated tissue and circulating concentrations of cytokines including interleukin-6 (IL-6) and tumor necrosis factor-α (TNFα). The origin of inflammation in renal disease is multifactorial, and involves reduced clearance of pro-inflammatory cytokines, reactive oxygen species and effects of comorbid conditions, such as diabetes (12). Inflammation is also a risk factor for MI and death in uremic patients (31) and a marker of severity and progression of heart failure (32). Experimentally, increased inflammatory cytokine mRNA expression is seen in the heart after renal ischemia-reperfusion injury (33). Dietary phosphate overload directly induces systemic increases in serum and tissue TNFα in a model of adenine-induced chronic renal failure (34). Conversely, renal macrophage infiltration and inflammatory cytokine expression is significantly increased from as early as 3 days post-MI (35). Activation of inflammasome pathways have been seen in both acute and CKD, with infiltrating macrophages specifically implicated (36). It is interesting to note that statins may reduce effects of inflammation in both the kidney and heart and may be protective (37).

##### Nitric oxide

The nitric oxide synthase (NOS) inhibitor asymmetrical dimethyl arginine (ADMA) accumulates in renal failure (38) and contributes to a reduction in NO systemically and intra-renally. NOS inhibition with N-nitro-l-arginine administration (l-NAME) depletes NO and both exacerbate renal dysfunction, and induce permanent cardiac dysfunction in rats with sub-total renal nephrectomy (39). Further evidence for the role of NO comes from those studies showing that chronic administration of l-NAME on its own also induces heart and kidney damage similar to that found in CRS (40). The model is characterized by a progressive increase in BP over 10 weeks with severe proteinuria, glomerulosclerosis, and tubulointerstitial fibrosis, and elevation in serum creatinine (40). Further, SNS activation is a recognized feature of the l-NAME model. Proteinuria and cardiac hypertrophy induced by chronic l-NAME treatment is abrogated by bilateral renal sympathetic denervation, but not hydralazine, even when blood pressure and NO depletion are equivalent (40).

##### Oxidative stress

Reactive oxygen species, produced as a result of redox reactions in various cells, have been recognized as key chemical mediators causing cellular damage and organ dysfunction in both CVD and CKD. There is growing evidence that oxidative stress is one of the central mediators of CRS. Increased oxidative stress is seen in patients with cardiac (41) and renal failure (42) and the reduced availability of NO impairs vasodilatation, and reduces renal perfusion (43).

It is now widely hypothesized that interactions between the RAAS system, the SNS and inflammation, may all potentiate CRS through excessive oxidative stress pathways (44). Consistent with this, experimental studies have identified several dysregulated pathways in heart failure and in CKD that lead to increased oxidative stress. Dahl salt-sensitive rats show increased LV NADPH oxidase activity, which is normalized by Angiotensin II blockade (45). In rats with sub-total nephrectomy, mitochondrial respiration in the heart is dysregulated, with cardiomyocytes isolated from uremic animals more susceptible to oxidant induced cell death than their normal counterparts (46).

##### Protein-bound toxins

Uremic toxins, such as indoxyl sulfate (IS), appear to accelerate the progression of CKD via profibrotic and oxidative pathways. Oral administration of IS in sub-totally nephrectomized rats induced renal tubular injury, renal interstitial fibrosis and glomerular sclerosis, leading to functional impairment (increased serum creatinine and blood urea nitrogen). These changes are associated with increased renal expression of profibrotic genes, such as TGFβ1, tissue inhibitor of metalloproteinases-1, and pro-collagen α1(I). Glomerulosclerosis and renal impairment has also been demonstrated in sub-totally nephrectomized rats receiving indole (47). The presence of IS, not indole, in the urine of indole-loaded animals confirms the protein metabolite hypothesis of IS production (47). IS has effects on cardiac myocytes and can cause cardiac hypertrophy and fibrosis (48, 49).

##### Neurohormonal disturbance

SNS over-activation is observed early in CKD, stemming from renal ischemia, raised angiotensin II levels, and suppression of NO amongst other causes. It is deleterious causing hypertension, LVH and eventually ventricular dysfunction and dilatation (12). Renal denervation in small animals can be achieved both surgically and chemically. These techniques have been widely used to study SNS activation and the relationship to hypertension in various experimental models of renal disease including sub-total nephrectomy (50) and deoxycorticosterone acetate (DOCA)-salt hypertension (51). Sympathectomy directly prevents onset and progression of albuminuria after chronic cardiac volume overload caused by aortic regurgitation (17).

##### Mineral bone disorder

The burden of excess CVD in patients with CKD is partly attributed to systemic disturbances in mineral metabolism and changes in bone histomorphometry. This is frequently accompanied by soft tissue calcification (52) especially within the arterial wall, where it is associated with significant mortality and morbidity (53). Patients with CKD also have a preponderance of medial arterial calcification (MAC), as well as greater calcification of intimal lesions. Pathophysiologically, aortic MAC is linked to alterations in vessel compliance, which exposes the heart to changes in vascular compliance and resistance changes in systolic pressure leading to LVH and myocardial fibrosis (54). Impaired aortic recoil results in lower diastolic pressures and a widened pulse pressure and reduced perfusion of coronary arteries, leading to sub-endocardial ischemia (55). Loss of vessel compliance may also impact on renal autoregulation (56). Although not a consistent finding in man (57), rodent models of renal failure demonstrate a convincing relationship between vascular stiffness and calcification scores (58, 59).

Three rodent animal models are commonly employed to study mineral bone disorder (MBD) and its cardiovascular sequelae in the context of CKD: 1) phosphate/vitamin D loading post 5/6 nephrectomy (60); 2) adenine-induced renal failure (61); and 3) the mouse electrocautery model of CKD (62) in strains with a genetic predisposition to vascular calcification (e.g., LDLR^−/−^ or apoE^−/−^). While these models consistently generate biochemical changes, calcification phenotypes are inconsistent due to differences in diet, study duration and the genetic background of the animals. Indeed, some inbred rodent strains (e.g., C57BL/6 mice and Sprague-Dawley rats) are surprisingly calcification resistant. Without concurrent calcitriol administration and or high phosphate feeding (>1% diet), vascular calcification after 5/6 nephrectomy is only apparent after 24 weeks (63). The mouse electrocautery model shows variable CKD, and mild hyperphosphataemia, even with high phosphate feeding. Interestingly, induction of CKD in transgenic atherosclerotic-prone animals only imparts a modest increase in aortic calcium relative to non-uremic littermates. Induction of milder renal impairment (equivalent to CKD Stage 2) by less intensive cautery of one kidney followed by contralateral nephrectomy (64) provides compelling evidence of the development of MBD in early CKD. The generation of uremic mice with adenine-enriched diets has gained considerable interest; here, animals develop advanced CKD, hyperphosphataemia (even without dietary phosphate loading), severe hyperparathyroidism despite normocalcaemia, and MAC within 4 weeks when on a 0.75% adenine diet (65, 66).

Crucially however, the MBD animal models discussed, thus far are generated by acute injury and rely on the consequent development of CKD. To date, few models of spontaneous CKD with well-characterized MBD and vascular calcification have been described. One such model, heterozygous Han:SPRD (Cy^/−^) rats (a model of autosomal dominant polycystic kidney disease) develop slowly progressive CKD, hyperphosphataemia, hyperparathyroidism and bone abnormalities but the vascular calcification generated is not progressive and only present in a subset of animals even after 38 weeks (67). Other models of spontaneous CKD (e.g., the Col4a3 null mouse model of human autosomal-recessive Alport syndrome), despite showing convincing biochemical evidence of CKD-MBD, do not exhibit a consistent vascular calcification phenotype (68).

A plethora of *in vitro* and *in vivo* studies have evoked the now widely accepted view that vascular calcification is a highly regulated and principally cell-mediated phenomenon that recapitulates many features of physiological ossification (69). There is strong evidence of osteochondrocytic differentiation of vascular cells in the calcified intimal plaques of high-fat fed LDLR^−/−^ and ApoE^−/−^ mice (70, 71), as well as in the arterial media of adenine and mineral stressed 5/6 nephrectomy models (72, 73). However, while de-differentiation of VSMC to a synthetic phenotype is found in some knockout models deficient in calcification inhibitors (e.g., matrix Gla protein (MGP)^−/−^ mice) (74), it is not a consistent finding (e.g., fetuin-A-deficient mice) (75). Moreover, mutations in the regulators of mineralization can manifest themselves quite differently in rodents and man. For instance, MGP^−/−^ mice have massive MAC. In humans, however, inactivating mutations in MGP (Keutel syndrome) exhibit infrequent arterial calcification (76). Conversely, inactivating mutations in Ennp1 encoding the pyrophosphate synthesizing ectoenzyme nucleotide pryophosphatase/phosphodiesterase results in the devastating syndrome, Generalized Arterial Calcification of Infancy (77). Ablation of homologous gene, *Npps*, in mice however, results in ossification of the spinal ligaments and peri-articular calcification but with relatively minor arterial involvement and only a modestly shortened lifespan (78).

Finally it is worth noting that despite considerable enthusiasm for the role of VSMC phenotype switching as a major mechanism for vascular calcification, the evidence for this phenomenon in human CKD is currently limited to a subset of patients on hemodialysis (79, 80). Indeed, even in this setting, evidence of such changes appear conspicuously absent at some vascular sites exclusively affected by medial calcification (81). An explanation for this variable disease penetrance is not currently forthcoming, although it should also be stressed that analyzes to date have been on small-to-medium sized muscular arteries that are generally free from intimal/atherosclerotic disease involvement and which may, therefore, not be representative of changes occurring in some larger elastic vessels (e.g., aorta). This may in part also explain the apparent disparity with findings in some animal models of uremia, where studies have mainly centered on changes in VSMC phenotype in the aorta and where phenotypic switching would appear to occur relatively early in disease progression (64).

## Cardiac and Renal Involvement in Systemic Disease (Type 5 CRS)

In many cases cardiac and renal pathologies are common to a system-wide perturbation. Relevant models include metabolic syndromes, such as hypertension, diabetes, obesity, liver disease, myeloma, lupus, and other autoimmune disease. Self-evidently these models align closely with the traditional risk factors seen in CVD, and reflect both chronic and acute causes.

### Chronic conditions

#### Hypertension

A number of experimental models of hypertension exist, including amongst others, inbred models of inherited primary hypertension [spontaneously hypertensive rat (SHR), Milan hypertensive rat, dahl salt-sensitive rat, and transgenic models over expressing renin (mRen2)]. While these consistently display cardiac pathologies, only some have parallel changes in renal function (82), with high pre-glomerular resistance relatively protective in selectively bred SHRs (83). Whilst inherited hypertension is a rare but recognized cause of hypertension in humans (82), in animal models correction of hypertension *per se* is not sufficient to prevent progression of experimental CRS (84).

#### Diabetes

Several models of diabetes have shown simultaneous cardiac and renal dysfunction including accelerated models, developed in an attempt to more closely mimic the human condition. The transgenic (mRen-2) rat overexpresses the murine renin gene (85) with elevated angiotensin II activity. Streptozotocin (STZ) induced beta-cell destruction in the mRen-2 rat is an established model of diabetes and its complications. The major advantage being that these rodents develop functional and structural pathology closely mimicking that seen in advanced human diabetic nephropathy (85) and cardiomyopathy (86). In a similar manner, SHR made diabetic with STZ replicate the confounding hypertension seen in diabetic nephropathy (87). However, despite a similar rise in blood pressure, unlike the diabetic mRen2 rat, these animals do not usually progress to renal failure.

STZ administration in the atherosclerotic (ApoE^−/−^) mouse model accelerates both diabetic renal pathology and atherosclerosis, a major risk factor for MI ischemia (88).

#### Obesity

Obese Zucker (OZ) rats (readily available commercially, and extensively studied) are characterized by mild glucose intolerance and peripheral insulin resistance similar to that found in humans with Type 2 diabetes. These abnormalities precede the development of albuminuria and glomerular injury and animals show a parallel deterioration in cardiac output and renal function (89). Ultrastructural studies have shown cardiomyopathy in both the OZ rat, and in mice with a similar mutation (db/db mouse) (90). A comprehensive analysis in the db/db mouse has shown that albuminuria/glomerulopathy and cardiac contractile dysfunction appear after 2–4 months of hyperglycemia (91).

### Acute conditions

#### Liver Disease

Both acute cholestatic and chronic fibrotic liver disease cause renal and cardiac dysfunction. Bile duct ligation causes acute and chronic renal (92) and cardiac dysfunction (93). Administration of carbon tetrachloride (CCl_4_) is a model of acute (or if repeated, chronic) hepatic failure causing widespread disruption to cardiac and renal function, although CCl_4_ generates oxidant injury directly in other organs (94). Other relevant experimental models include toxin induced murine models, as well as some murine models of autoimmune hepatitis and primary biliary cirrhosis (95).

#### Lupus

The classic murine model of lupus (as a paradigm for other autoimmune disease) includes genetically predisposed crosses (New Zealand Black crosses) and toxin (pristane) induced models (96). Nevertheless, many of the murine models fail to fully replicate the multisystem manifestations of human lupus, and whilst most replicate lupus nephritis, other organs like skin and arthritis are inconsistently affected, although the NZB murine model does seem to develop pericardial, epicardial and myocardial inflammation (97). Because the murine model is not altogether representative of the human condition, other models including canine and porcine models have been developed (98, 99).

## Compound Effect of Combining Renal and Cardiac Pathologies

A less common experimental scenario looks at the effect of overlaying renal impairment on cardiac disease, and the reverse. For example sub-total nephrectomy accelerates pathological cardiac remodeling post-MI when performed 4 weeks after infarction with worse ejection fraction in those animals with renal impairment (100). Similarly when insults are reversed e.g., performing a sub-total nephrectomy a week before MI, LV damage is worse and associated with worse creatinine clearance (101) and renal blood flow, and more proteinuria and glomerulosclerosis. Sub-total nephrectomy followed by MI once renal injury is firmly established leads to more pronounced damage in both organs (102).

In sub-totally nephrectomized rats, temporary ligation of the descending branch of the left coronary artery after 3 weeks resulted in a larger area of cardiac necrosis (devoid of mitochondrial oxidation) than sham ligated paired controls. Infarcts after coronary artery ligation (CAL) were larger in animals with even modest renal impairment (84). A major disadvantage of these models is that experimental mortality is high. Although the effect is mild, reducing renal mass by uninephrectomy is also often used as a means of accelerating renal disease, but this is not in itself sufficient to produce a cardiac phenotype (103).

## Conclusion

No animal model in isolation reproduces the complexity of different CRS (Table 1). Nevertheless, animal models have provided valuable insights into the pathogenesis of CRS in all its forms (5). Like their counterpart clinical trials, animal studies have highlighted the mechanistic importance of non-traditional risk factors. To this end the RAAS system, SNS, indirect and direct effects of the uremic toxins, anemia, inflammation, neurohormonal factors and disturbances in mineral handling and bone turnover are all implicated causatively. However, although the small animal models frequently employed in these studies are readily amenable to further genetic manipulation and intervention, induction of injury is generally acute and unphysiological, and these models often fail to faithfully recapitulate the pathological features of human CRS.

**Table 1:** **Animal models of cardiorenal and renocardiac syndromes, and relative advantages and disadvantages of each**.

Model	Technique	Mechanism	Advantages	Disadvantages	Reference
**PRIMARY CARDIAC DISEASE**					
Coronary artery ligation (CAL)	Surgical	Myocardial ischemia (MI)	Widely used, well characterized	Variable renal pathology	(13, 35)
Aortic regurgitation	Surgical	Cardiac volume overload		Mild renal pathology	(16, 17)
**PRIMARY RENAL DISEASE**					
Sub-total nephrectomy (SNx)	Surgical	Uremia, renal insufficiency	Relevant to CKD in general, well characterized	Highly variable if not performed uniformly	(21)
**SIMULTANEOUS CARDIAC AND RENAL DISEASE**					
SNx followed by CAL	Surgical			High mortality	(101)
SNx followed by CAL with established CKD	Surgical		Clinical relevance		(102)
CAL followed by SNx	Surgical			High mortality, poorly characterized	(100)
CAL and uninephrectomy	Surgical		Lower mortality than SNx, and more reproducible	Unrepresentative of chronic kidney disease	(103)
Anthracycline antitumor antibiotics (e.g., adriamycin)	IV injection	Toxicity	Simple. Simultaneous cardiac and renal pathologies	Off target toxicity, cardiac and renal dose responses differ	(29)
		Anemia	Simple	Mild anemia	(29)
L-NAME	IV injection	NOS inhibition, SNS activity	Simultaneous cardiac and renal pathologies		(40)
**MODELS OF SYSTEMIC DISEASE WITH CARDIAC AND RENAL PATHOLOGIES**					
Spontaneously hypertensive rat (various inbred strains)	Spontaneous	Hypertension, RAAS		Uncommon cause of human hypertension	(82)
Zucker rat (inbred rat strain with leptin receptor deficiency)	Spontaneous	Dyslipidemia	Approximates type 2 diabetes	Leptin receptor mutations are rare in humans	(89)
db/db mouse (leptin receptor mutation)	Spontaneous	Dyslipidemia	Approximates type 2 diabetes	Leptin receptor mutations are rare in humans	(91)
Diabetic mRen2 rat (STZ diabetes in transgenic renin overexpressing rat)	Spontaneous, IV injection	hyperglycemia, hypertension, RAAS	Accelerated type 1 diabetes, simultaneous cardiac and renal functional changes	Hypertension is primary rather than secondary to diabetes	(85)
Lupus	Spontaneous			Unrepresentative of human condition	(96)
Hepatic bile duct ligation	Surgical			Off target pathology	(92, 93)
**BONE MINERAL DISORDERS**					
Phosphate/vitamin D loading post-SNx	Surgical, oral intake	Mineral bone disorder	Widely used, well characterized	Slow, high mortality, poorly reproducible with complications, unphysiological	(60)
Adenine	Oral intake	Mineral bone disorder	Simple, rapidly progressive	Substantial weight loss (dehydration)	(61)
Electrocautery in LDLR^−/−^ or apoE^−/−^ mice	Surgical, spontaneous	Mineral bone disorder	Good models of atherosclerosis	Not widely available, poor models of arteriosclerosis (lack of intimal calcification).	(62)
Han:SPRD^+/−^	Spontaneous	Mineral bone disorder	Spontaneous, mimics chronicity of process	Not widely available, no bone phenotype, mild calcification phenotype	(67)

*CAL, coronary artery ligation; l-NAME, N-nitro-l-arginine administration; NOS, nitric oxide synthase; MI, myocardial infarction; RAAS, renin angiotensin aldosterone syndrome; SNX, sub-total nephrectomy*.

In this article, we have discussed a range of models that can be used to mimic the mechanisms of human renal and cardiac disease, and examined how representative they are of the human condition. While not perfect, careful and ethical use of animal models offers the opportunity to examine the complex interactions seen in CRS in an accelerated time frame.

## Conflict of Interest Statement

The authors declare that the research was conducted in the absence of any commercial or financial relationships that could be construed as a potential conflict of interest.
